# Brazil and the Framework Convention on Tobacco Control: Global Health Diplomacy as Soft Power

**DOI:** 10.1371/journal.pmed.1000232

**Published:** 2010-04-20

**Authors:** Kelley Lee, Luiz Carlos Chagas, Thomas E. Novotny

**Affiliations:** 1 Centre on Global Change and Health, London School of Hygiene & Tropical Medicine, London, United Kingdom; 2 Independent Researcher – Public Health and Trade Policies, London, United Kingdom; 3 Graduate School of Public Health, San Diego State University, San Diego, California, United States of America

## Abstract

As part of the *PLoS Medicine* series on Global Health Diplomacy, Kelley Lee and colleagues provide a case study of Brazil's growing influence in international relations and global health, using as an example that country's role and use of “soft power” in the negotiation of the Framework Convention on Tobacco Control.

**Summary Points**

- “Soft power” is a diplomatic approach to obtain an objective through persuasion and collaboration, rather than through economic influence or political domination.
- Brazil's growing influence in international relations, as one of the so-called BRIC (Brazil, Russia, India, and China) countries, has been due to its effective use of soft power in key foreign policy negotiations.
- Brazil has shown soft-power leadership in negotiations concerning climate change, trade liberalisation, energy policy, nuclear non-proliferation, and recent health-related diplomatic activities. Policy consistency was shown in Brazil's constitutional guarantee of access to anti-retroviral drugs for people living with HIV/AIDS that required steadfast negotiations to ensure access within World Trade Organization guidelines.
- During negotiations for a Framework Convention on Tobacco Control (FCTC), Brazil demonstrated commitment to global health diplomacy by serving as an exemplar for domestic tobacco control, engaging in coalition politics, and providing leadership throughout the negotiation process.
- Brazil's influential role in the negotiation of the FCTC can be seen as an example of how global health has become a focus of soft power.

*This article is part of the* PLoS Medicine *Global Health Diplomacy series.*

## Introduction

One of the key developments in international relations during the early twenty-first century is the ascendance of the BRIC countries (Brazil, Russia, India, and China) (see Box 1 for terms and definitions). While their rising status stems largely from their demographic and economic growth (together accounting for about 40% of the world's population and 40% of global GDP [1]), also important has been what international relations scholars refer to as the growing use of “soft power.” The term “soft power” was coined by Joseph Nye during the 1990s to describe “how power is changing in world politics” since the end of the Cold War. He argued that, while military force and conquest remain important, power derived from technology, education, and economic growth have increased in significance. The result has been “a general diffusion of power” to a broader range of state and non-state actors. Given that “the solutions to many current issues of transnational interdependence will require collective action and international cooperation,” Nye argued that governments must use an appropriate balance of “soft power” (co-option and attraction) and “hard power” (coercion and payment) when pursuing their interests [2].

**Box 1. Terms and Definitions**

**BRIC countries:** An acronym referring to the fast-growing developing economies of Brazil, Russia, India, and China. The term was coined by investment bank Goldman Sachs in 2001 in its predictions that, by 2050, the four economies would together eclipse those of the current richest countries.

**Agreement on Trade Related Intellectual Property Rights (TRIPS):** An international trade agreement administered by the World Trade Organization (WTO) that sets out minimum standards for intellectual property regulation. Signed in 1994, and coming into effect in January 1995, the agreement sets out requirements that member states meet on such matters as copyright, patents, trademarks, geographical indications (a name or sign used on certain products which corresponds to a specific geographical location or origin) and industrial design.

**Antiretroviral drugs (ARVs):** Medications for the treatment of infection by retroviruses, namely HIV/AIDS. Affordable access to such drugs has been the subject of intense global debate because of patent protections asserted by pharmaceutical companies under TRIPS and other trade agreements.

**Framework Convention on Tobacco Control (FCTC):** An international treaty negotiated under the auspices of WHO that sets out minimum standards for national, regional, and international tobacco control measures, including the setting of broad limits on tobacco production, sale, distribution, advertisement, taxation, and government policies. Signed in 2003, the treaty came into force in February 2005. The treaty currently has 168 state parties.

This paper examines the process by which Brazil asserted influence in the negotiation of the Framework Convention on Tobacco Control (FCTC) as an example of soft power. Implemented under the bylaws of the World Health Organization (WHO) [3], the FCTC has been the product of multi-level and multi-actor negotiation processes that define “global health diplomacy” [4]–[6]. A fuller understanding of Brazil's contribution to the FCTC provides insights into the pursuit of global health cooperation alongside broader foreign policy objectives, as well as the emerging practice of global health diplomacy.

## Methodology

As part of a broader project on “The tobacco industry, public policy and global health” and our case study of the FCTC and global health diplomacy, the authors sought to obtain views of Brazil's role in the FCTC negotiations. The authors carried out key informant interviews with Brazilian policy makers, diplomats, and public health advocates on the country's role in FCTC negotiations from December 2008 through January 2009. Interviews were conducted by LCC in Portuguese, transcribed, and translated. Triangulation of reported perceptions was achieved through a literature review of primary and secondary sources including government reports and Web sites, industry documents, reports by nongovernmental organizations, and unpublished research dissertations.

This research was approved by the London School of Hygiene & Tropical Medicine Ethics Committee as part of the US National Institutes of Health-funded “Tobacco control, public policy and global health” project (Application No. 5612). In addition, interview quotes were approved by the relevant key informants for citation.

## Brazil's New Prominence in Global Health

Brazil has become increasingly prominent in international relations in recent years through its leadership in climate change [7], trade, energy policy, and nuclear nonproliferation negotiations [8]. By combining economic growth with progressive domestic social policies, the country has defied orthodox thinking on development. It has been in the realm of global health, however, that Brazilian diplomacy has been particularly noteworthy, beginning with negotiations on access to medicines for treatment of HIV/AIDS. Because of its constitutional requirement for equity in access to antiretroviral (ARV) therapy [9], and the political will to address the issue, Brazil successfully confronted and negotiated a satisfactory resolution to barriers imposed on drug availability by the Agreement on Trade-Related Aspects of Intellectual Property Rights (TRIPS). With the US government aligning with powerful corporate interests, Brazil's championing of free and universal access to ARVs earned worldwide respect among public health advocates [10]. While other countries, such as Thailand and South Africa, also sought to challenge the pharmaceutical industry on restrictive pricing policies, as Nunn and colleagues argue, Brazil became the first developing country to offer free ARV treatment to HIV/AIDS patients despite claims by the World Bank that such a policy was not cost-effective [11]. Importantly, the country has seen a dramatic decline in AIDS-related morbidity and mortality as a result of its treatment program, a success story that has served as a role model for the expansion of global support for HIV/AIDS treatment in other countries. In this way, Brazil helped bridge a chasm between public health and trade policy through its national HIV/AIDS policy [12].

## Brazilian Tobacco Control Policy as an Exemplar

Brazilian leadership was critical to the successful conclusion of the FCTC negotiations in 2003. Following the establishment of a model national tobacco control program, Brazilian medical doctor and former coordinator of the National Tobacco Control Programme, Vera Luiza da Costa e Silva, was recruited to lead WHO's Tobacco Free Initiative (TFI), and Brazilian diplomats were appointed to chair the Intergovernmental Negotiating Body (INB) for the FCTC. A fuller understanding of Brazil's contribution to the FCTC process may provide lessons about the conduct of global health diplomacy in other contexts.

Brazil's National Tobacco Control Programme implemented many innovations: Brazil was the second country (after Canada) to adopt graphic warnings on cigarette packages, the first to create a body to regulate tobacco contents and emissions, and the first to ban the use of “light” and “mild” terms in describing tobacco products. According to an interview with Tania Cavalcante, Executive Secretary of the National Inter-ministerial Commission to Implement the FCTC, Brazil promoted these advances in many INB negotiation sessions, and encouraged other countries to support them as treaty elements. Importantly, Brazil's status as one of the biggest producers and exporters of tobacco, while at the same time achieving high visibility in tobacco control, provided additional credibility for its leadership role in the FCTC negotiations [13]. As diplomat Frederico Duque Estrada Meyer, former assistant to Ambassadors Celso Nunes Amorim and Luiz Felipe de Seixas Correa , put it, “Some countries have restrictive anti-smoking policies like Brazil, but are not producers. Others, are big producers but with a very liberal tobacco policy….we were leading on both sides….we represented both conflicting interests.” In our interviews, the Brazilian former Director of the TFI, Vera Luiza da Costa e Silva, further emphasized this complex negotiating position:

*To be a big producer, a big exporter with a strong and influential industry, and a big consumer market for tobacco products, with pressures in the domestic market generated by allies of a powerful industry, Brazil actively supported all the WHO resolutions that led to the creation of the Intergovernmental Negotiating Body. To be a country subject to all these factors and also able to implement tobacco control, we were talking at that time of being a model for other countries, mainly for developing countries. We were sending a message that, under any circumstances, a government committed to this priority, despite the weight of other factors, could still have one of the best tobacco control programs in the world and support and adopt a treaty on tobacco control.* (Translated from Portuguese)

## Coalition Diplomacy: Bringing Together Public Health and Foreign Policy

Brazil's ability to grapple with the diversity of interests at the national level, including a powerful tobacco industry, began with the establishment of the Inter-Ministerial National Commission on the Control of Tobacco Use in 1999. Backed by the highest levels of government, the Commission was a consultative body to determine the official government position on the FCTC negotiations. Importantly, nine ministries were represented on the Commission, including Inland Revenue, Trade and Development, and Agriculture [14]–[15]. This commission, including all pertinent stakeholders, ensured that tobacco control was embodied in consistent policies throughout government and not only as a health ministry issue. The close involvement of the Ministry of Foreign Affairs, in particular, backed by the highest levels of government, ensured a clear and unified endorsement of health goals within Brazilian foreign policy:

*The participation of the Ministry of Foreign Affairs in Geneva clearly signaled, largely to tobacco industry representatives, that the Government was cohesive in its position against smoking. The Government's stance dispelled any doubt that the negotiations could only be about health interests*. (Translated from Portuguese) [Interview with Ambassador Santiago Alcazar, former Manager of Social Issues Unit, Ministry of Foreign Affairs]

This was an approach that protected governmental negotiation positions from the vested interests of the tobacco industry, and it can be considered one strategy for the implementation of Article 5.3 of the FCTC on the protection of public health policies with respect to tobacco control from commercial and other vested interests. Once negotiations commenced, the government extended coalition building to civil society organizations (CSOs), which, through participation in health councils at the federal, state, and municipal levels, mobilised to implement tobacco control interventions [13]. Their role proved particularly critical in supporting its subsequent ratification by the Brazilian Senate after the signing of the FCTC by the Chief Executive.

The need to build a broad domestic coalition on tobacco control across government, civil society, and the public health community was heightened by the industry's own strategic lobbying of related economic interests to help it oppose stronger binding obligations of the FCTC. As described in an internal document of British American Tobacco (BAT), released to the public in the 1990s as a result of US litigation [16]:

*[W]e know how the FCTC will be negotiated and we know what countries will be involved. All end markets have been alerted and key political and legal arguments have been distributed….British American Tobacco's response to date has consisted of attempting to engage in dialogue with the WHO, running a lobbying campaign based on legal and political arguments designed to preserve adults freedom to smoke, maintain our ability to trade freely and to raise awareness of the FCTC's implications among finance, trade, agriculture and employment ministers around the world. We have had some success in some countries but it is by no means complete.* [17]

Brazil is cited by the industry as among the key countries where such a strategic approach was needed.

Faced with this industry threat, Brazil then extended its coalition building to the regional and global levels. In addition to formal FCTC negotiations, informal meetings were held, according to Calvacante, as “a strategy adopted by chairs of different working subgroups when there was an impasse and consensus could not be reached.” Brazil played an active part in many of these meetings, especially at the regional level, she said: “The objective was to start sowing regional consensus before the INB negotiations to speed up the process. We organised the first meeting for the Americas region.”. At the same time, CSO activity was organised through the Framework Convention Alliance (FCA), a worldwide coalition of nongovernmental organizations and interested parties, which played an important contributory role in FCTC negotiations, ratification, and implementation [18]. As Alcazar writes, “[d]ifferent groups in civil society come together as an interested party in the process of implementing an international treaty. It is as if civil society, as an interested party—and certainly an unstructured one—becomes a player on the international stage” [13].

## Brazilian Leadership in Global Negotiations

A strategically important decision by the WHO TFI was the appointment of Celso Nunes Amorim, then Brazil's Permanent Representative to the United Nations and other international organizations in Geneva, as INB Chair. Amorim was recognised as a skilled and experienced diplomat, particularly during his tenure as negotiator in UN talks on disarmament, trade, and security. The US delegation described him as “a steady hand and [providing] good leadership” [19]. When Amorim became Ambassador to the United Kingdom in 2002, he was succeeded as INB Chair by another experienced diplomat, Luiz Felipe de Seixas Correa. Along with skilful diplomats, Brazil was enabled by the strong support of the Minister of Health, José Serra, who recognized that the international negotiating process had direct effects on Brazilian national tobacco control efforts and public health, according to Vera Luiza da Costa e Silva.

As an emerging economy, Brazilian support for the FCTC was important for countering industry-led arguments that tobacco control was a “first world issue.” Despite epidemiological evidence to the contrary [20], the industry claimed that

*the first world, Anglo-Saxon and English speaking political economies, ... are fuelling the debate and in many cases driving the political agenda within the WHO. Most third world countries have other priorities but are not able to resist the pace, drive and political dynamics which are moving the FCTC forward.* [21]

To counter such claims, the TFI sought to build support within the developing world. The six deputy chairs of the INB to lead specific working groups—the US, Australia, Iran, India, South Africa, and Turkey—were carefully selected to ensure both developed and developing country representation and to encourage regional activism. The Southeast Asia Tobacco Control Alliance (SEATCA), formed in 2001, played a similar role. In Latin America, regional meetings were held to build consensus within such groups as the Group of Latin America and Caribbean Countries (GRULAC) and Mercosur (Mercado Común del Sur):

*Group meetings of this nature happen regularly in Geneva and are opportunities to discuss a diversity of themes, which are discussed in a diplomatic context. As Brazil was chairing the treaty negotiations, it had a privileged forum to amplify the relevance and importance of what the WHO was proposing.* (Translated from Portuguese) [Interview with Vera Luiza da Costa e Silva]

Brazil then used its diplomatic channels to build linkages across regions:

*They not only performed their role during the meetings, but also took advantage of meetings with representatives of other countries and regions at their respective permanent missions in Geneva to disseminate information about the contents and scope of the treaty, especially about the necessity of countries to give priority to this public health subject in parallel with the “great star” in the city which was the World Trade Organization.* (Translated from Portuguese) [Interview with Vera Luiza da Costa e Silva]

The result of this effort was effective expanded participation by developing countries in the negotiations:

*Those developing countries, which were under assault by massive tobacco industry marketing and political pressure campaigns, have fought back in Geneva, and the strengthening of the treaty during this last round of negotiations is a tribute to their courage and persistence in resisting the efforts by the United States, Germany and Japan to weaken the treaty and water down crucial clauses. Developing countries formed a strong alliance with NGOs and championed our positions during the negotiations.* [22]

## Conclusions

Brazil's leadership in global health diplomacy must be understood as part of the country's political and economic ascendance in international relations. As the world's tenth largest economy, and an integrated member of the world trading system, the country's influence over a wide range of global health issues is likely to grow in coming decades. Brazil has recognised that traditional practices of hard power can be inappropriate in a globalized world. Its understanding of soft power, in the form of normative leadership and the use of “opinion-shaping instruments” [23], suggests that a new kind of diplomacy is emerging to achieve collective action on shared challenges such as global health. Through its principled stance on ARVs, and its domestic commitment to strong and effective tobacco control, Brazil has earned widespread credibility as a diplomatic leader. This, in turn, has helped to reinforce domestic policy on tobacco control. Brazil's remarkable example also suggests that engagement in health diplomacy is increasingly seen as a core component of what it means to be a global citizen [24].

## Abbreviations

ARV: antiretroviral
BAT: British American Tobacco
BRIC: Brazil, Russia, India, and China
CSO: civil society organization
FCA: Framework Convention Alliance
FCTC: Framework Convention on Tobacco Control
GRULAC: Group of Latin America and Caribbean Countries
INB: Intergovernmental Negotiating Body
SEATCA: Southeast Asia Tobacco Control Alliance
TRIPS: Trade-Related Aspects of Intellectual Property Rights
WHO: World Health Organization
WTO: World Trade Organization
